# Expression of Thrombospondin-1 Modulates the Angioinflammatory Phenotype of Choroidal Endothelial Cells

**DOI:** 10.1371/journal.pone.0116423

**Published:** 2014-12-30

**Authors:** Ping Fei, Ismail Zaitoun, Mitra Farnoodian, Debra L. Fisk, Shoujian Wang, Christine M. Sorenson, Nader Sheibani

**Affiliations:** 1 Department of Ophthalmology and Visual Sciences, University of Wisconsin School of Medicine and Public Health, Madison, Wisconsin, United States of America; 2 Department of Pediatrics, University of Wisconsin School of Medicine and Public Health, Madison, Wisconsin, United States of America; 3 McPherson Eye Research Institute, University of Wisconsin School of Medicine and Public Health, Madison, Wisconsin, United States of America; 4 Department of Biomedical Engineering, University of Wisconsin School of Medicine and Public Health, Madison, Wisconsin, United States of America; Center for Cancer Research, National Cancer Institute, United States of America

## Abstract

The choroidal circulation plays a central role in maintaining the health of outer retina and photoreceptor function. Alterations in this circulation contribute to pathogenesis of many eye diseases including exudative age-related macular degeneration. Unfortunately, very little is known about the choroidal circulation and its molecular and cellular regulation. This has been further hampered by the lack of methods for routine culturing of choroidal endothelial cells (ChEC), especially from wild type and transgenic mice. Here we describe a method for isolation and culturing of mouse ChEC. We show that expression of thrombospondin-1 (TSP1), an endogenous inhibitor of angiogenesis and inflammation, has a significant impact on phenotype of ChEC. ChEC from TSP1-deficient (TSP1−/−) mice were less proliferative and more apoptotic, less migratory and less adherent, and failed to undergo capillary morphogenesis in Matrigel. However, re-expression of TSP1 was sufficient to restore TSP1−/− ChEC migration and capillary morphogenesis. TSP1−/− ChEC expressed increased levels of TSP2, phosphorylated endothelial nitric oxide synthase (NOS) and inducible NOS (iNOS), a marker of inflammation, which was associated with significantly higher level of NO and oxidative stress in these cells. Wild type and TSP1−/− ChEC produced similar levels of VEGF, although TSP1−/− ChEC exhibited increased levels of VEGF-R1 and pSTAT3. Other signaling pathways including Src, Akt, and MAPKs were not dramatically affected by the lack of TSP1. Together our results demonstrate an important autocrine role for TSP1 in regulation of ChEC phenotype.

## Introduction

The choroid is a thin, highly vascularized and pigmented tissue positioned under the sensory retina that forms the posterior portion of the uveal tract (the iris, cilliary body, and choroid). The choroid plays an important role in retinal homeostasis and functions to dissipate heat, and nourish the retinal pigment epithelial cells and outer retinal photoreceptor cells [1]. Abnormalities in this vasculature result in many congenital and adult diseases such as choroidal coloboma and age-related macular degeneration [2]–[4]. The choroidal endothelium plays a critical role in pathologic conditions, such as choroidal effusion, inflammation, neovascular membrane and neovascularization of choroidal melanoma [5]–[7]. Although much is known about retinal endothelial cells (EC), as well as endothelial cells from vascular bed of other tissues, choroidal EC (ChEC) have not been well studied.

Vascular EC from various tissues display a broad functional and phenotypic heterogeneity as well as showing organ specificity [8]. Unlike retinal EC, ChEC have fenestrations, through which the nutrients are readily transported to the RPE and photoreceptors. In addition, ChEC are shown to differ in their response to various growth factors including vascular endothelial growth factor (VEGF), fibroblast growth factor (FGF2), and insulin-like growth factor-1 (IGF-1) compared to retinal EC [9]–[13]. However, the detailed underlying mechanisms remain poorly understood. The ability to culture ChEC from human, bovine, and ovine [14]–[17] has been very helpful in providing insight into the physiology of these cells as well as their cell autonomous regulatory mechanisms. Understanding of the regulatory mechanisms and how their alterations contribute to choroidal vascular dysfunction is critical for treatment of many diseases with a neovascular component including AMD.

It is difficult to obtain a pure ChEC culture because these cells are strongly embedded in the choroidal tissue and are surrounded by various other cell types that often contaminate the culture. To our knowledge, only primary bovine, human, and ovine ChEC have been isolated and cultured, be it with a limited proliferative capacity [18]–[21]. There are no reports of isolation and culture of ChEC from mouse eyes. As an important component in the process of vasculogenesis and angiogenesis, the biology of mouse vascular cells has been a recent focus of many studies. Mice offer the added benefits of well-established genetic modification techniques. Many genetically modified mouse strains have been established in the past two decades. Studies on the effect of certain single or multiple genetic modifications have revealed an advanced understanding of their roles in many basic biological processes.

Thrombospondin-1 (TSP1) is a member of the matricellular family of TSP proteins with potent anti-angiogenic and anti-inflammatory activity. TSP1 inhibits angiogenesis in vivo and EC proliferation and migration in vitro [22], [23]. In contrast, TSP1 is an important autocrine factor for vascular smooth muscle cells’ proliferation and migration [24]. We have shown that mice deficient in TSP1 (TSP1−/−) exhibit increased retinal vascular density. This was mainly attributed to the failure of the developing retinal vasculature to undergo appropriate pruning and remodeling in the absence of TSP1 [25]. Furthermore, we showed that over expression of TSP1 in the eye results in the attenuation of retinal vascular development and ischemia-mediated neovascularization [26]. Therefore, appropriate expression of TSP1 plays an essential role in retinal vascular homeostasis. However, the role TSP1 plays in choroid vascular development and neovascularization remains unknown.

We recently showed that mice deficient in TSP1 exhibit enhanced choroidal neovascularization in the laser-induced choroidal neovascularization model [27]. This was mainly attributed to enhanced recruitment of macrophages into the site of laser burns, consistent with the ocular anti-inflammatory proposed role for TSP1 [28]. In addition, patient with neovascular AMD demonstrated decreased expression of TSP1 in Bruch’s membrane preparations [29], [30]. However, the cell autonomous function of TSP1 in ChEC remains unknown.

The ability to culture EC has been instrumental in developing assays to study the mechanisms, which impact angiogenesis and vascular cell phenotypes. Here we describe a method for the isolation and propagation of mouse ChEC from wild type (TSP1+/+) and TSP1−/− immortomice. Furthermore, we demonstrate that these cells can be readily expanded, retaining their EC markers, and can aid in defining the functional consequences of gene targeting on EC properties. We showed that ChEC prepared from TSP1−/− mice were less proliferative, less migratory, and more apoptotic compared with TSP1+/+ cells. Lack of TSP1 resulted in attenuation of ChEC capillary morphogenesis and adhesion to various ECM proteins. Furthermore, the enhanced eNOS phosphorylation, and increased NO levels were observed in TSP1−/− ChEC. The TSP1−/− ChEC also showed a significant increase in expression of inflammatory mediator iNOS, a major source of NO and oxidative stress. Thus, expression of TSP1 in ChEC has a significant impact on their angioinflammatory phenotype, and its altered production may contribute to pathogenesis of exudative AMD.

## Materials and Methods

### Ethics Statement

All experiments were carried out in accordance to the Association for Research in Vision and Ophthalmology Statement for the Use of animals in Ophthalmic and Vision Research and were approved by the Institutional Animal Care and Use Committee of the University of Wisconsin School of Medicine and Public Health.

### Experimental Animals

Immortomice expressing a temperature-sensitive SV40 large T antigen were obtained from Charles River Laboratories (Wilmington, MA). Thrombospondin-1 deficient (TSP1−/−) mice in the C57BL/6J background were generated as previously described [31]. TSP1−/− mice were crossed with immortomice, previously backcrossed to C57BL/6 mice for at least 10 generations, and the immorto-TSP1−/− mice were identified by PCR analysis of DNA isolated from tail biopsies. The PCR primer sequences were as follows: immorto-forward: 5′-CCT CTG AGC TAT TCC AGA AGT AGT G-3′, immorto reverse: 5′-TTA GAG CTT TAA ATC TCT GTA GGT AG-3′; Neo-forward: 5′-TGC TCT CCA TCT GCA CGA GAC TAG-3′, Neo-reverse: 5′GAG TT GCT TGT GGT GAA CGC TCA G-3′; TSP1-forward: 5′-AGG GCT ATC TGG AAT TAA TAT CGG-3′, and TSP1-reverse: 5′-GAG TTT GCT TGT GGT GAA CGC TCA G-3′.

### Preparation of Anti- PECAM-1 Antibody Coated Magnetic Beads

Sheep anti-rat Dynabeads (Dynal Biotech, Lake Success, NY) were washed three times with serum-free DMEM (Dulbecco’s Modified Eagle’s Medium; Invitrogen, Carlsbad, CA) and then incubated with rat anti-mouse PECAM-1 monoclonal antibody MEC13.3 (BD Pharmingen, San Diego, CA) overnight at 4°C (10 µl beads in 1 mL DMEM). Following incubation, beads were washed three times with DMEM containing 10% fetal bovine serum (FBS) and resuspended in the same medium, and stored at 4°C.

### Isolation and Culture of Choroidal EC

Eyes from one litter (6 to 10 pups) of 4-week-old TSP1+/+ and TSP1−/− immortomice were enucleated and all connective tissue and muscle was removed from the sclera. Under a dissecting microscope in cold DMEM, the anterior eye was removed, followed by the lens, vitreous, retina and optic nerve, leaving only a tissue composed of RPE, choroid and sclera. These tissues (8 to 14 from one litter) were pooled together, rinsed with DMEM, minced into small pieces in a 60 mm tissue culture dish using sterilized razor blades, and digested in 5 ml of collagenase type I (1 mg/ml in serum free DMEM, Worthington, Lakewood, NJ) for 45 min at 37°C. Following digestion, DMEM with 10% FBS was added and cells were pelleted. The cellular digests then were filtered through a double layer of sterile 40 µm nylon mesh (Sefar America Inc., Fisher Scientific, Hanover Park, IL), centrifuged at 500×g for 10 min to pellet cells, and cells were washed twice with DMEM containing 10% FBS. The cells were resuspended in 1 ml medium (DMEM with 10% FBS), and incubated with sheep anti-rat magnetic beads pre-coated with anti-PECAM-1 as described above. After affinity binding, magnetic beads were washed six times with DMEM with 10% FBS and bound cells in endothelial cell growth medium were plated into a single well of a 24 well plate pre-coated with 2 µg/ml of human fibronectin (BD Biosciences, Bedford, MA). Endothelial cells were grown in DMEM containing 10% FBS, 2 mM L-glutamine, 2 mM sodium pyrovate, 20 mM HEPES, 1% non-essential amino acids, 100 µg/ml streptomycin, 100 U/ml penicillin, freshly added heparin at 55 U/ml (Sigma, St. Louis, MO), endothelial growth supplement 100 µg/ml (Sigma, St. Louis, MO), and murine recombinant interferon-γ (R & D, Minneapolis, MN) at 44 units/ml. Cells were maintained at 33°C with 5% CO_2_. Cells were progressively passed to larger plates, maintained, and propagated in 1% gelatin-coated 60 mm dishes.

### FACS Analysis

Monolayers of choroidal EC on 60 mm culture dishes were washed once with PBS containing 0.04% EDTA, and incubated with 3 ml of cell dissociation solution (TBS; 20 mM Tris-HCl, 150 mM NaCl pH 7.4 containing 2 mM EDTA and 0.05% BSA) to collect the cells from the plate. Cells were washed once with DMEM containing 10% FBS, and blocked in 0.5 ml TBS with 1% goat serum for 20 min on ice. Cells were pelleted, resuspended in 0.5 ml of TBS with 1% BSA containing an appropriate dilution of primary antibody (as recommended by the supplier), and incubated on ice for 30 min. For vascular EC markers, cells were incubated with anti-PECAM-1, anti-endoglin (all from BD BioSciences), anti-VE-cadherin (Santa Cruz, Santa Cruz, CA), and FITC-conjugated B4-lectin. For intracellular detection cells were fixed with 0.5 ml of 2% paraformaldehyde and 0.1% Triton-X-100 in TBS for 15 min on ice, washed with TBS containing 1% BSA, and incubated with primary antibodies (prepared in 0.5 ml TBS with 1% BSA, 0.1% Triton X-100) for 30 min on ice. For integrin expression analysis, anti-α1-integrin (BD Biosciences), α_2_-, α_3_-, α_5_-, α_v_-, β_1_-, β_8_-integrin (Santa Cruz), and β_3_-, α_5_β_1_-, α_v_β_3_-integrin (Millipore) antibodies were used. Anti-CD36, VCAM-1, ICAM-1, ICAM-2 (BD Biosciences), CD47 (eBiosciences), VEGF-R1, VEGF-R2 (R&D Systems), and fenestration markers Pan-End also called MECA-32 or PV-1 (BD Biosciences), HARE-M20 (sc-27752), and HARE-Y20 (sc-27751; Santa Cruz Biotechnology) also called stabilin-2, were also used. Following incubation with primary antibody, cells were washed twice with TBS containing 1% BSA, and then incubated with appropriate FITC-conjugated secondary antibody (Pierce, Rockford, IL; 1∶200 dilution in 0.5 ml of TBS with 1% BSA for 30 min on ice). The stained cells were washed twice with TBS containing 1% BSA, resuspended in 0.5 ml of TBS with 1% BSA, and analyzed by FACScan caliber flow cytometer (Becton-Dickinson).

### Cell Proliferation

Cell proliferation assay was performed by plating cells in 60 mm tissue culture dishes. The cell numbers were counted every other day in triplicate for 12 days. 1×10^4^ cells were plated on gelatin-coated 60 mm tissue culture dishes, and the cells were counted the next day for day one. Cell were then fed every other day and counted on the days that they were not fed for 12 days. The rate of DNA synthesis was measured using Click-iT-EdU Alexa Fluor 488 kit (Invitrogen) as recommended by the supplier. The assay measures incorporation of EdU (5-ethynyl-2′-deoxyuridine), a nucleoside analogue of thymidine, during cell proliferation. TSP1+/+ and TSP1−/− choroidal EC (5×10^5^) were plated on 60 mm tissue culture and incubated with 10 µM EdU in culture medium for 3 h at 33°C. The DNA synthesis was analyzed by measuring incorporated EdU using FACSscan caliber flow cytometry (Becton-Dickinson).

### Apoptosis and Cell Viability

Apoptosis was determined by measuring caspase activation using Caspase-Glo 3/7 assay system (Promega, Madison, WI) as recommended by the supplier. The assay provides caspase-3/7 DEVD-aminoluciferin substrate and the caspase 3/7 activity is detected by luminescent signal. For the assay, choroidal EC (5×10^3^) from TSP1+/+ and TSP1−/− were plated in 96 well plates. As an apoptotic stimulus, choroidal EC were incubated with 1 mM hydrogen peroxide (H2O2, Fisher Scientific, Fair Lawn, NJ) or 1 µM staurosporine (Invitrogen) in EC growth medium for 8 h at 33°C. The caspase activity was detected by a luminescent microplate reader (Victa2 1420 Multilabel Counter, PerkinElmer, Waltham, MA).

Cellular viability of ChEC was demonstrated by MTS assay (Promega). Plated ChEC (4×10^3^) on 96 well plates were incubated with different concentrations of H_2_O_2_ (0–2 mM) for 2 days at 33°C, and incubated further with MTS solution for 3 h. The viability was determined by measuring absorbance at 490 nm using a microplate reader (Thermomax, Molecular Devices, Sunnyvale, CA) and determined as percentage of control untreated cells. All samples were prepared in triplicates and repeated twice.

### Indirect Immunofluorescence

Cells (1×10^5^) were plated on gelatin-coated glass coverslips until confluent (1–2 days), washed in PBS, fixed and permeabilized with 4% PFA/0.1% Triton X-100 for 10 min in room temperature. Slides were washed with PBS and incubated with anti-VE-cadherin, N-cadherin, β-catenin (BD Bioscience), ZO-1 (Zymed, Carlsbad, CA), vinculin (Sigma), and FITC-conjugated phalloidin (Sigma) in TBS containing 1% BSA at 37°C for 40 min. After washing with TBS, cells were incubated with appropriate Cy3-conjugated secondary antibody (1∶1000 dilution in TBS containing 1% BSA) at 37°C for 40 min. Cells were washed with TBS five times and analyzed using a fluorescent microscope (Carl Zeiss Optical Inc., Germany) and images were captured in digital format.

### Scratch Wound Assays

Cells (6×10^5^) were plated in 60 mm tissue culture dishes and allowed to reach confluence (2–3 days). Cell monolayers were wounded with a 1 ml micropipette tip, rinsed with DMEM containing 10% FBS twice, and fed with EC growth medium containing 1 µM 5-fluorouracil (Sigma) to exclude potential contribution of cell proliferation to wound closure. The concentration of 5-fluorouracil used here was determined by dose studies and selected based on inhibition of DNA synthesis and lack of toxicity (not shown). The wound closure was monitored and photographed at 0, 24, and 48 h using a phase microscope in digital format. For quantitative assessment, the distances migrated as percent of total distance were determined as described previously [32].

### Transwell Migration Assays

The transwell migration assay was conducted as previously described [32]. Briefly, the bottom of Costar transwells with 8 µM pore size (Fisher) were coated with fibronectin (2 µg/ml PBS) at 4°C overnight. After rinsing with PBS, the bottom side of the transwell was blocked with 2% BSA in PBS for 1 h at room temperature. Choroidal EC were trypsinized, resuspended in serum-free DMEM, and 1×10^5^ cells in 0.1 ml was added to top of the transwell membrane. Cells were incubated for overnight at 33°C, fixed with 2% paraformaldehyde (PFA) for 10 min at room temperature, and stained with hematoxylin/eosin. The stained membranes were mounted on a glass slide and the number of migrated cells through the membrane, which attached to the bottom, was determined by counting 10 high-power fields (×200).

### Cell Adhesion to Various Extracellular Matrix Proteins

Cell adhesion assays were performed in 96 well flat-bottom plates (Nunc Immunoplate Maxisorp, Fisher Scientific) coated with various concentrations of matrix proteins, or BSA as control. Fibronectin, vitronectin, collagen I, and collagen IV (BD Biosciences) were serially diluted in TBS containing 2 mM CaCl2 and 2 mM MgCl2 (TBS with Ca/Mg) and coated (50 µl) the wells of the 96 well plates overnight at 4°C. Plates were washed four times with 200µl of TBS with Ca/Mg containing 1% BSA at room temperature for 1 h. Cells were removed from the tissue culture plates using 3 ml of cell dissociation buffer (2 mM EDTA, 0.05% BSA in TBS), washed with TBS, and resuspened in cell binding buffer (20 mM HEPES, 150 mM NaCl, 4 mg/ml BSA, pH 7.4) at approximately 6×10^5^ cells/ml. The plates were washed once with 50 µl of TBS with Ca/Mg, and 50 µl of cell for 2 h at 37°C in a humidified incubator. After incubation, the plates were washed with 200 µl of TBS with Ca/Mg to remove non-adherent cells until no cells were left in the BSA coated wells. For quantification of the number of adherent cells, the levels of intracellular acid phosphatase were measured by lysing the adherent cells in 100 µl of lysis buffer (50 mM sodium acetate pH 5.0, 1% Triton X-100, 6 mg/ml p-nitrophenyl phosphate) and incubating at 4°C overnight. The reaction was neutralized by adding 50 µl of 1 M NaOH and the absorbance was determined at 405 nm using a microplate reader (Thermomax, Molecular Devices). All samples were done in triplicates and repeated twice.

### Western Blot Analysis

Cells (6×10^5^) were plated on 60 mm culture dishes and allowed to reach approximately 90% confluence. The cells were then rinsed once with serum free DMEM, and incubated with EC growth medium without serum for 2 days. Conditioned medium (CM) was collected and centrifuged to remove cell debris. The cells were also lysed in 0.1 ml of lysis buffer (50 mM HEPES pH 7.5, 100 mM NaCl, 0.1 mM EDTA, 1 mM CaCl2, 1 mM MgCl2, 1% Triton X-100, 1% NP-40, and protease inhibitor cocktail (Roche Biochemicals, Mannheim, Germany). To detect phospho-eNOS, cells were serum starved for 2 days and stimulated with serum containing medium for 30 min. Following incubation, cells were rinsed with cold PBS containing 1 mM Na3OV4 (twice), and lysed in 0.1 ml of lysis buffer containing 3 mM Na3OV4 and 5 mM NaF. Protein concentrations were determined using BCA protein assay (Biorad, Hercules, CA), sample were adjusted for protein content, mixed with appropriate volume of 6x SDS-sample buffer, and analyzed by SDS-PAGE (4–20% Tris-glycine gels, Invitrogen). Proteins were transferred to nitrocellulose membrane and the membrane was blocked with blocking buffer (0.05% Tween-20 and 5% skim milk in TBS). Anti-tenascin-C (Millipore; AB19013), TSP1 (A6.1, Neo Markers, Fremont, CA), Endoglin (Cymbus Biotechnology), iNOS, nNOS (BD Biosciences), fibronectin (sc-9068), NOS (sc-648), STAT3 (H-190; sc-7179) and Src (sc-18; Santa Cruz Biotechnology), phospho-Src, Akt, phospho-Akt, phospho-STAT3, phospho-eNOS, p38, phospho-p38, ERKs, phospho-ERKs (Cell Signaling), JNK, phospho-JNK, osteopontin,TSP2 (R&D systems), and β-actin (Sigma) antibodies were diluted to 1∶1000 in blocking buffer and incubated with the membrane for 2 h at room temperature. Blots were washed with TBST (TBS with 0.05% Tween-20) and incubated with appropriate secondary HRP-conjugated antibody. The blots were then washed with TBST and developed using ECL (Fisher Scientific). The blot was stripped and incubated with anti-β-actin (Sigma) antibody for loading control.

### Capillary Morphogenesis Assays

Tissue culture plates (35 mm) were coated with 0.5 ml of Matrigel (10 mg/ml, BD Biosciences) and allowed to harden by incubating at 37°C for 30 min. Cells were removed by trypsin EDTA, washed with DMEM containing 10% FBS, and resuspended at 1×10^5^ cells/ml in EC growth medium without FBS. Cells (2×10^5^) in 2 ml were applied to the Matrigel-coated plates, incubated at 33°C, photographed after 18 h using a Nikon microscope in a digital format. For quantitative assessment of the data, the mean numbers of branch points were determined by counting the number of branch points in five high-power fields (×100). Longer incubation times did not further improve the degree of capillary morphogenesis.

### Ex Vivo Sprouting of RPE-Choroid Complex

Choroidal explants were prepared and cultured as described previously [33], with some modifications. Briefly, postnatal day 21 (P21) mice were anesthetized using isoflurane and killed by cervical dislocation. Eyes were enucleated, washed three times and kept in ice-cold DMEM medium. Attached tissues to the outer surface of the eyeball (blood vessels, fatty and connective tissues) were shaved in ice-cold DMEM medium and under the dissection microscope. The cornea, lens and corpus vitreum were removed before the intermediate segment containing the sclera, choroid, retinal pigment epithelium (RPE) and the retina was dissected along the whole circumference. The neuroretina and sclera were then removed, and choroid and the RPE were sectioned into 0.5- to 1.0 mm pieces. These pieces were finally transferred into 35 mm culture dishes coated with 0.5 ml of Matrigel (10 mg/ml) (BD Biosciences). Preparations were transferred into a 37°C cell culture incubator without medium for 20 minutes to solidify Matrigel. Endothelial cell growth medium was then added (2 ml/dish) and incubated at 37°C with 5% CO2 for eight days. Explants were fed once every 48 h. After 8 days, preparations were fixed with 4% PFA for 30 min at room temperature, washed three times in 1xPBS before they were imaged using a Nikon microscope. Area of sprouting was measured and analyzed using Image J software (National Institute of Health, Bethesda, MD). The mean sprouting area was determined from area/pixel intensity of ten explants per eye that were prepared and cultured in a single dish. At least 3 mice per genotype were used for these experiments.

### Re-Expression of TSP1 in TSP1−/− ChEC

To express TSP1 in TSP1−/− ChEC cells, 2.5×10^5^ cells were plated in 35 mm tissue culture dishes. The next day, adenoviruses encoding TSP1 or GFP (375 pfu/cell, 30 µl) were mixed and diluted in 500 µl of opti-MEM (Life Technologies) and incubated for 15 min at room temperature. Following incubation, the tissue culture plates were washed twice in serum-free DMEM and incubated with 0.5 ml of adenovirus and adeno booster mixture overnight. The next day, medium containing virus and booster mixture were removed and fresh medium containing 10% FBS was added to the plates and incubated for 3 days before they were used for further analysis.

### Intracellular NO Measurements

The intracellular NO level of TSP1+/+ and TSP1−/− choroidal EC was determined using DAF-FM diacetate (Invitrogen). DAF-FM diacetate is a cell-permeable molecule, which passively defuses into the cell and becomes deacetylated by intracellular esterases forming DAF-FM. DAF-FM fluorescence increases significantly after it reacts with NO and can be detected using a fluorescein filter. Cells (5×10^3^ cells/0.1 ml) were plated on gelatin-coated 96-well black/clear bottom plates (BD Falcon) and incubated overnight. The next day, medium was removed; fresh EC growth medium containing 30 µM DAF-FM diacetate (0.1 ml/well) was added, and incubated for 40 min. Following incubation, the medium was removed and replaced with fresh EC growth medium without DAF-FM (0.1 ml/well). The samples were incubated for 30 min, washed with TBS (0.1 ml/well; twice), and DAF-FM fluorescence of cells in TBS was detected at an excitation of 485 nm and an emission of 535 nm using a fluorescent microplate reader (Victa2 1420 Multilabel Counter, PerkinElmer). These assays were performed three times in triplicate and results were normalized for cell number.

### Secreted VEGF Measurements

The amount of secreted VEGF produced by TSP1+/+ and TSP1−/− choroidal EC was determined using Mouse VEGF Immunoassay kit (R&D system). Cells (6×10^5^) were plated on 60 mm tissue culture dishes and allowed to reach approximately 90% confluence. The cells were then rinsed once with serum free DMEM and incubated with 2 ml of EC growth medium without serum for 2 days. The CM was centrifuged to remove cell debris and the secreted VEGF in CM was analyzed according to manufacturer’s instruction. The amount of VEGF was determined using a standard curve generated with known amounts of VEGF in the same experiment.

### Statistical Analysis

Statistical differences between control and treated samples were evaluated with student’s unpaired *t*-test (2-tailed) or two-way ANOVA with Bonferroni correction for multiple comparisons when appropriate. Mean ± SEM are shown. P values ≤0.05 were considered significant.

## Results

### Isolation and Characterization of TSP1+/+ and TSP1−/− ChEC

**Figure 1:**
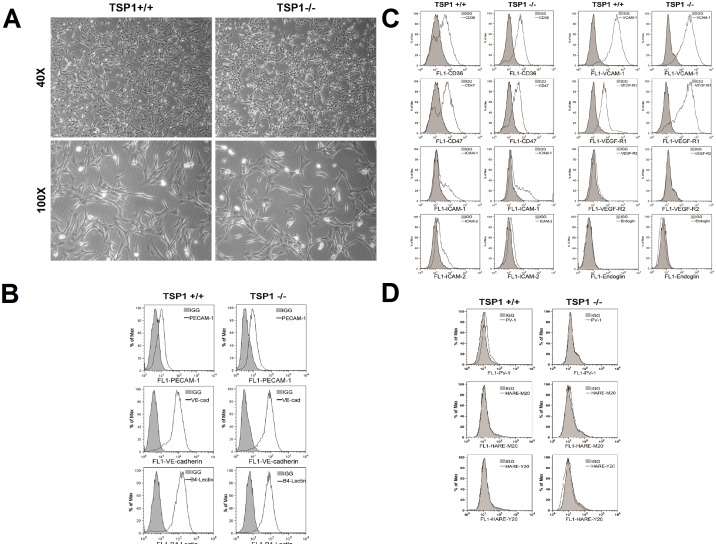
Isolation and characterization of mouse choroidal endothelial cells (ChEC). Thrombospondin1 (TSP1)+/+ and TSP1−/− ChEC were prepared as described in MATERIALS AND METHODS and cultured on gelatin-coated plates in 60-mm dishes. A: cells were photographed in digital format at ×40 and ×100 magnification. Note TSP1−/− ChEC exhibited a similar elongated and spindly morphology compared with TSP1+/+ ChEC. B: The expression of vascular EC markers in ChEC. ChEC were examined for expression of PECAM-1, VE-cadherin (VE-cad), and B4 lectin by FACS analysis. Shaded areas show control IgG staining. Note the similar expression of these cellular markers in both cells. C: FACS analysis for expression of other cell surface markers. Please note expression of CD36, CD 47, ICAM-1, ICAM-2, and VCAM-1 expression in these cells. We also detected significant expression of VEGF-R1 in these cells whose level was increased in TSP1−/− ChEC. The VEGF-R2 expression was almost undetectable. D: FACS analysis of EC markers for fenestration, PV-1 and HTAR (stabilin-2). Please note minimal expression of these markers. These experiments were repeated at least twice with two different isolations of choroidal EC, with similar results.

Successful isolation and culture of mouse choroidal EC has not been previously reported. The ability to culture ChEC has allowed us to directly study the cell autonomous role of TSP1 in modulation of ChEC properties. Using TSP1+/+ and TSP1−/− immortomice, we have successfully isolated and determined the proangiogenic and proinflammatory characteristics of ChEC. ChEC were first released from choroid tissues by incubating with collagenase type I, and selectively separated from contaminating cells using magnetic beads pre-coated with anti-PECAM-1, an EC marker. The magnetic beads coated cells were then plated in a single well of a 24-multiwell plate coated with fibronectin and allowed to reach confluence. The cells were passed to 2 wells of a 24- multiwell plate and then to a 60 mm tissue culture dish. This resulted in isolation of a homogeneous population ChEC with greater than 98% purity determined by FACS analysis and immunofluorescence staining. Fig. 1A shows the morphology of ChEC prepared from TSP1+/+ and TSP1−/− mice. TSP1−/− ChEC exhibited a similar elongated and spindly morphology compared with TSP1+/+ ChEC, when plated on gelatin-coated plates.

We next determined the expression of EC markers in these cells by FACS analysis (Fig. 1B). Lack of TSP1 did not affect the expression level of PECAM-1, VE-cadherin and B4-lectin in ChEC. However, endoglin expression was quite low in TSP1+/+ ChEC, and TSP1−/− ChEC expressed almost no endoglin (Fig. 1C). These results were further confirmed by Western blot analysis (not shown). Fig. 1C,D shows expression of other markers including CD36, CD47, ICAM-1, ICAM-2, VCAM-1, VEGF-R1, VEGF-R2 and endoglin, as well as markers of fenestration PV-1 and HARE (stabilin-2) with minimal staining. TSP1-deficiency minimally affected the expression of these markers, with the exception of VEGF-R1 whose level was increased in TSP1−/− ChEC. The growth of these cells at the non-permissive temperature, or with longer passage (up to 25 passages) at permissive temperature, minimally affected the expression of these markers, as we previously reported with other retinal cells (not shown [34]–[37]).

### Alterations in Cell-Cell Interactions

**Figure 2:**
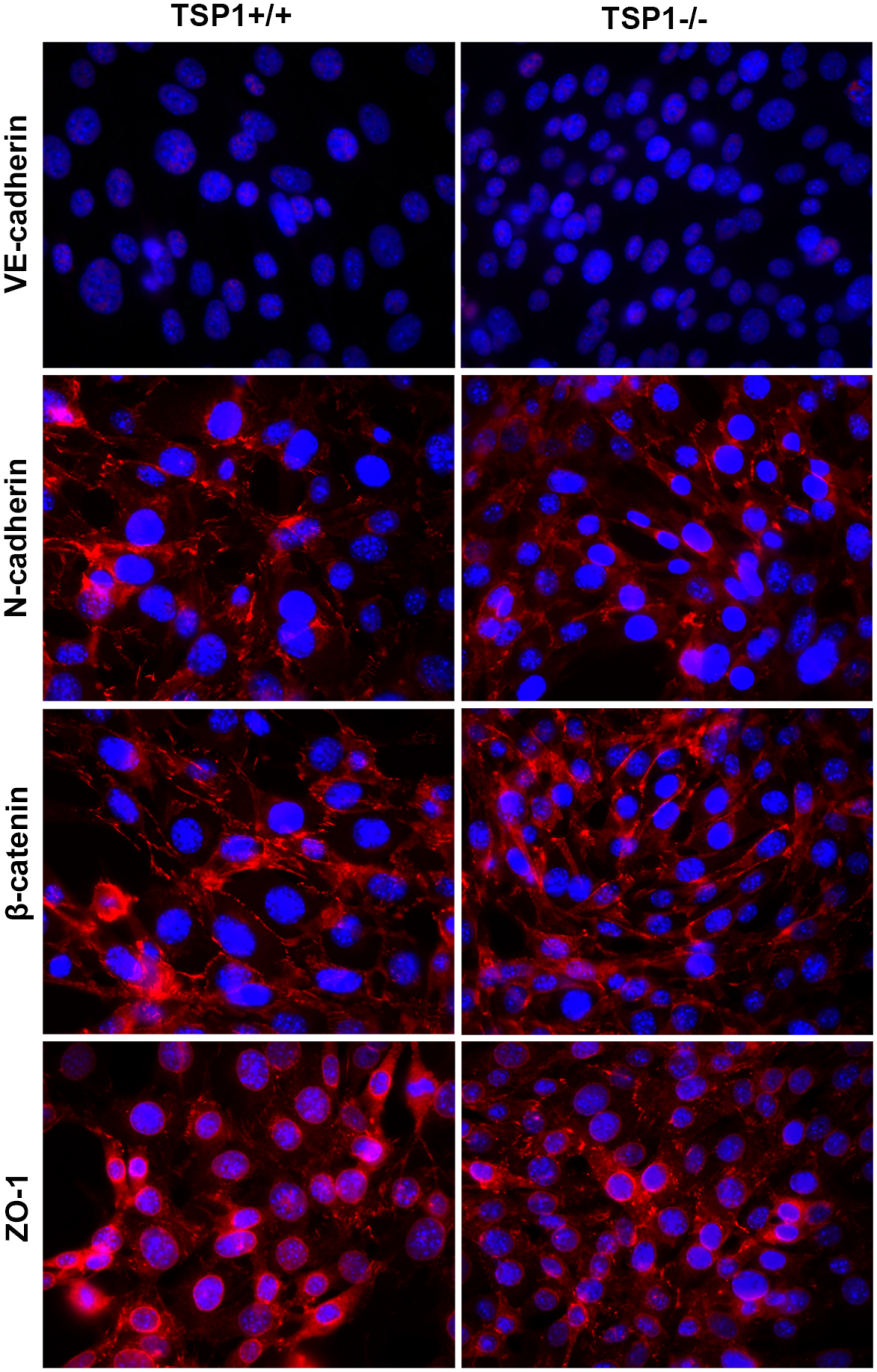
Cellular localization and expression level of VE-cadherin, N-cadherin, β-catenin, and ZO-1. A: TSP1+/+ and TSP1−/− ChEC were grown on fibronectin-coated coverslips to confluence and stained as described in Methods with specific antibodies. No staining was observed when primary antibody was left out. Please note VE-cadherin showed no staining in both TSP1+/+ and TSP1−/− ChEC. N-cadherin, β-catenin had similar levels and junctional localization in TSP1+/+ and TSP1−/− choroidal EC. ZO-1 showed similar perinuclear localization and punctate junctional localization in both TSP1+/+ and TSP1−/− ChEC. B: Western blot analysis of junctional proteins. Consistent with immunofluorescence staining, no VE-cadherin protein was detectable in ChEC. Similar levels of N-cadherin, β-catenin, and ZO-1 were detected in ChEC. These experiments were repeated at least twice with two different isolations of choroidal EC, with similar results.

VE-cadherin mediates cell-cell interactions through formation of adherens junctions, which are important for maintaining vascular integrity [38]. We examined expression and localization of VE-cadherin by indirect immunofluorescence staining of TSP1+/+ and TSP1−/− ChEC. Despite significant expression of VE-cadherin on the surface of these cells by FACS (Fig. 1B), no VE-cadherin junctional localization was observed in the ChEC regardless of the TSP1 status (Fig. 2A), even though retinal EC showed junctional localization of VE-cadherin under identical conditions (not shown; [34], [39]). Perhaps another cadherin may participate in formation of adherens junctions in ChEC.

N-cadherin is a member of the cadherin family of proteins with important roles in angiogenesis and vascular stabilization [40], [41]. VE-cadherin competes with N-cadherin for formation of adherens junctions in EC, and generally localizes to the site of cell-cell contact [42]. We next determined expression and localization of N-cadherin in TSP1+/+ and TSP1−/− ChEC. A similar level of N-cadherin and junctional localization was observed in TSP1+/+ and TSP1−/− ChEC (Fig. 2A, B). This is in contrast to retinal EC where VE-cadherin is the predominant junctional cadherin [34], [43].

The localization of β-catenin, another component of adherens junctions, was not affected in TSP1−/− ChEC. The β-catenin staining showed a punctate staining pattern in both TSP1+/+ and TSP1−/− ChEC (Fig. 2A, B). Another protein with important function in formation of tight junctions is ZO-1, whose junctional localization in EC is VE-cadherin dependent. ZO-1 showed similar perinuclear localization and punctate staining pattern at sites of cell-cell contact in TSP1+/+ and TSP1−/− ChEC (Fig. 2). Thus, lack of TSP1 did not have a significant impact on expression and localization of ChEC junctional proteins, although their localization was different from that observed in retinal EC [39].

### TSP1−/− ChEC Grow at a Slower Rate and Exhibit Increased Levels of Apoptosis

**Figure 3:**
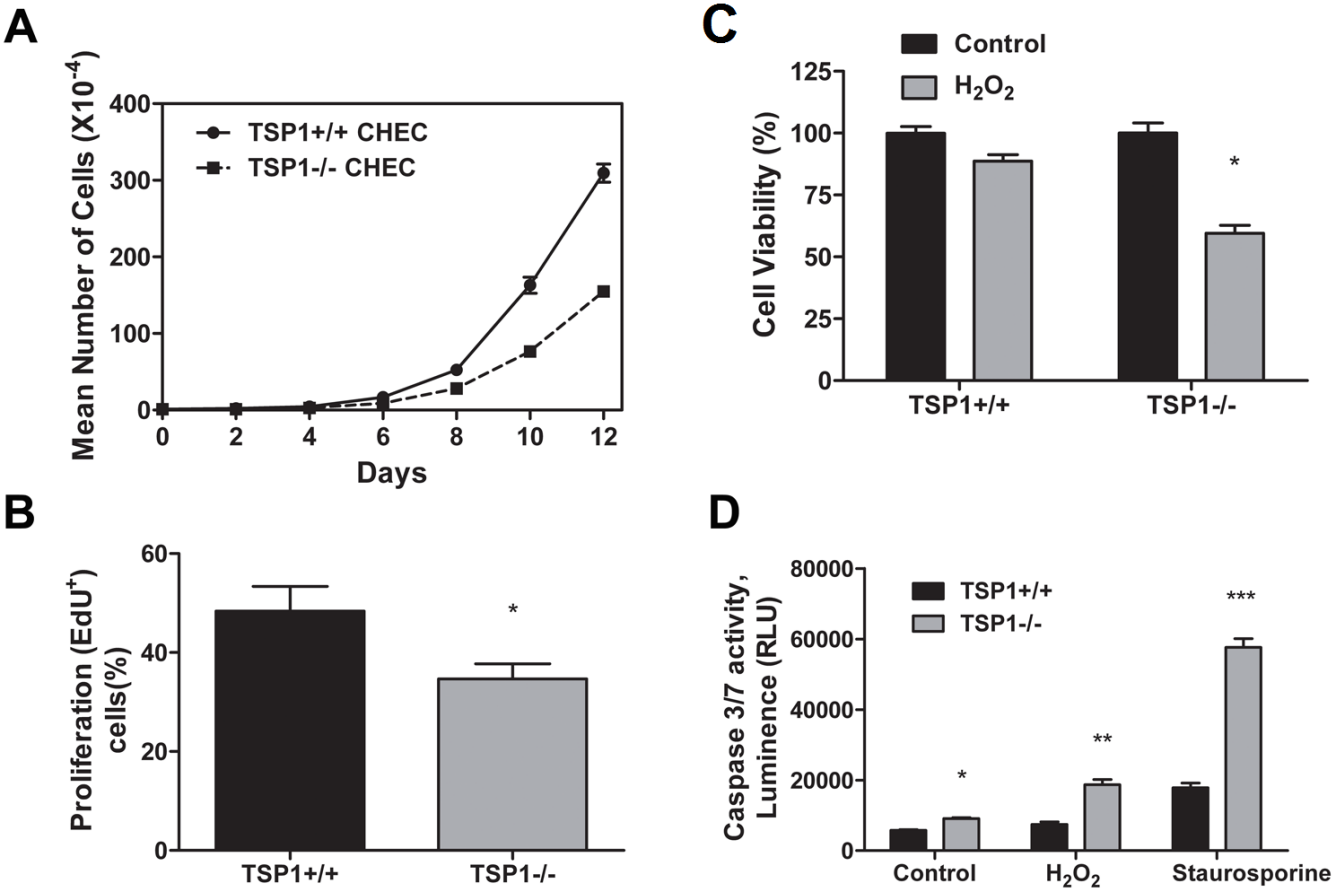
Altered proliferation and apoptosis of TSP1−/− ChEC. A and B: the rate of ChEC proliferation was determined by counting the number of cells in triplicate after different days in culture as described in Methods (A) and by analyzing the rate of DNA synthesis by FACScan flow cytometry analysis (B; P<0.05; *n* = 3). C: Hydrogen peroxide (H_2_O_2_) toxicity of ChEC was measured by MTS assay. ChEC were incubated with 1 mM H_2_O_2_ in EC growth medium for 2 days in 96-well plates and subjected to the MTS assay. TSP1−/− ChEC were significantly more sensitive to cytotoxic effect of H_2_O_2_ (*P<0.05; *n* = 3). D: The rate of apoptosis was determined by measuring caspase activity with luminescent signal from caspase-3/7 DEVD-aminoluciferin substrate, as recommended by the supplier. As an apoptotic stimulus, H_2_O_2_ and staurosporine in EC growth medium were added for 8 h. Please note the significant increase in the rate of apoptosis in TSP1−/− ChEC compared with TSP1+/+ cells (*,**,*** *P<*0.05; *n* = 3). RLU, Relative Light Unit.

The effect of TSP1 deficiency on the growth rate of ChEC was determined by counting the number of cells for 12 days. Fig. 3A shows a significant decrease in the growth rate of TSP1−/− ChEC compared with TSP1+/+ cells. At the 12th day of culture, the cell number for TSP1−/− ChEC was 50% of the TSP1+/+ ChEC (*P*<0.05; *n* = 3). To determine whether the decreased growth rate was due to a decrease in rate of DNA synthesis, we measured the percentage of cells undergoing active DNA synthesis by labeling with EdU, a synthetic nucleoside analog. TSP1−/− ChEC showed a decreased level of DNA synthesis compared with TSP1+/+ ChEC (Fig. 3*B*; *P*<0.05; *n* = 3).

The cytotoxicity of H_2_O_2_ toward ChEC was evaluated with the MTS cytotoxicity assay. TSP1+/+ and TSP1−/− ChEC were plated on gelatin-coated 96-well plate and incubated with different concentrations of H_2_O_2_ for 2 days. Cell viability was decreased in a concentration-dependent manner in both TSP1+/+ and TSP1−/− ChEC, such that at 2 mM H_2_O_2_ we observed a 90% decrease in viability of both cell types (not shown). Incubation with 1 mM H_2_O_2_ decreased viability of TSP1+/+ ChEC by 11%, while that of TSP1−/− ChEC was decreased by 40% (Fig. 3*C*; *P<*0.05; *n* = 3). Thus, TSP1−/− ChEC were more sensitive to H_2_O_2_-mediated cytotoxicity compared with TSP1+/+ ChEC.

We next determined the level of apoptosis in TSP1+/+ and TSP1−/− ChEC under steady-state culture conditions. Apoptotic cell death was determined by evaluation of the activation status of caspase 3/7. TSP1−/− ChEC showed a 1.6-fold increase in the rate of apoptosis compared with TSP1+/+ ChEC (Fig. 3*D*; *P*<0.05; *n* = 3). H_2_O_2_, a highly reactive oxygen species, is a potent inducer of apoptosis in EC. We determined the level of H_2_O_2_-induced caspase 3/7 in TSP1+/+ and TSP1−/− ChEC. The ChEC were incubated with 1 mM H_2_O_2_ in culture medium for 8 h. H_2_O_2_-induced apoptosis in TSP1−/− ChEC was increased 2.5 times compared with TSP1+/+ ChEC (Fig. 3*D*). Similar results were observed with staurosporine, a known inducer of apoptosis (1 µM; Fig. 3*D*; *P*<0.05; *n* = 3). Thus, the decreased growth was attributed to a decreased level of DNA synthesis and increased level of apoptosis in TSP1−/− ChEC.

### TSP1−/− ChEC Were Less Migratory

**Figure 4:**
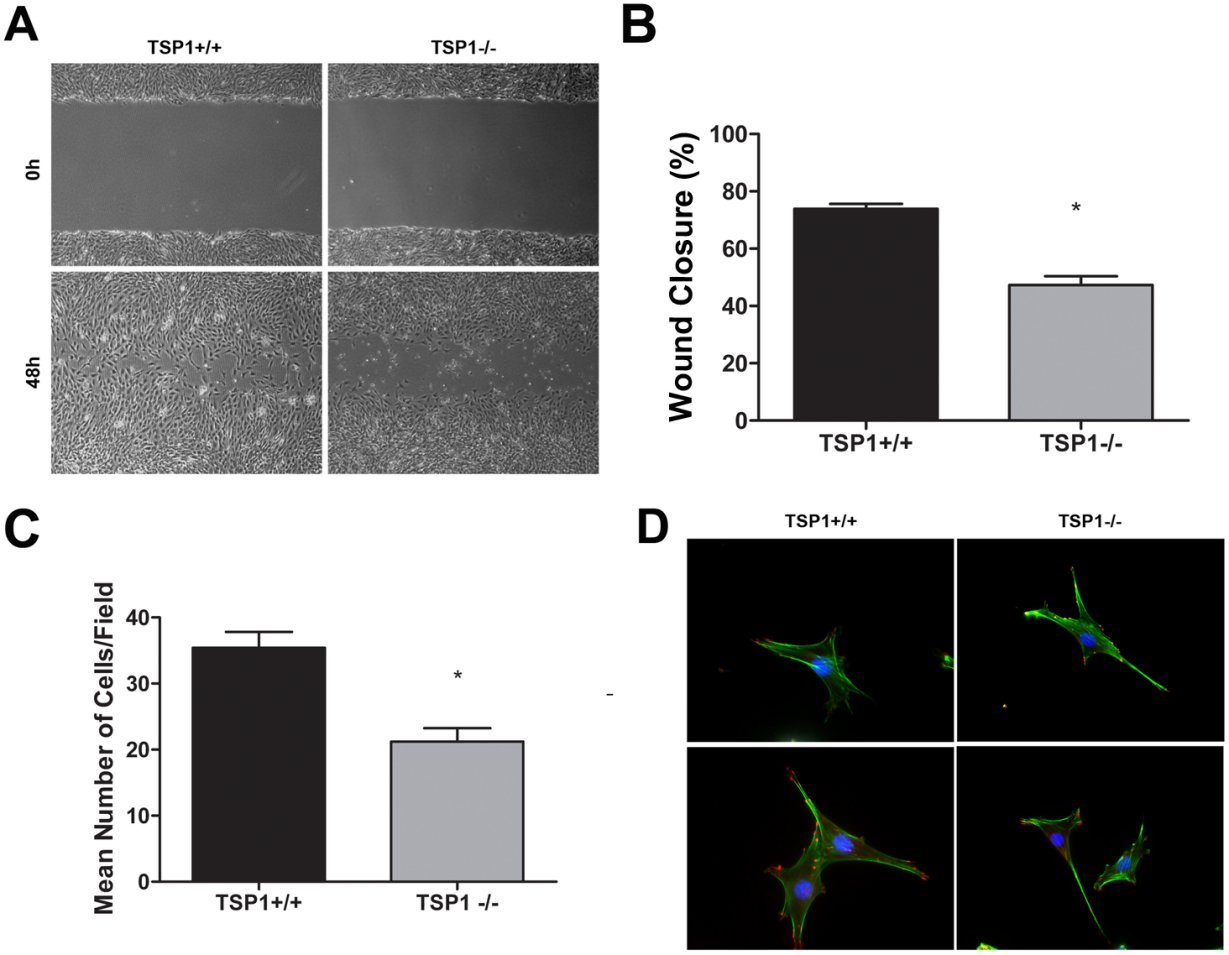
TSP1−/− ChEC are less migratory. A: Cell migration was determined by scratch wound assay of the ChEC monolayers on gelatin-coated plates. Wound closure was monitored by photography within 48 h. B: Quantitative assessment of the data (*P<0.05, *n* = 3). *C*: Cell migration was also determined using a transwell migration assay (*P<0.05, *n* = 3). *D*: The indirect immunofluorescence staining of phalloidin (green; actin) and vinculin (red; focal adhesions). Please note similar actin stress fibers and focal adhesion organizations in TSP1+/+ and TSP1−/− ChEC. The quantitative assessment of fluorescence intensities showed no significant differences (*P*>0.05; n = 3; not shown). These experiments were repeated with two different isolations of cells with similar results.

Cell migration is fundamental to the ability of EC to undergo capillary morphogenesis during angiogenesis. A scratch wound assay was performed to investigate the migratory properties of ChEC. Confluent monolayers of TSP1+/+ or TSP1−/− ChEC were wounded, and wound closure by cell migration was monitored with still photography. To eliminate the impact of cell proliferation on migration and wound closure these experiments were performed in the presence of a low concentration of 5-fluorouracil (1 µM). Wound closure was significantly delayed in TSP1−/− ChEC by 48 h compared with TSP1+/+ ChEC (Fig. 4A). The quantitative assessment of the data is shown in Fig. 4B (*P*<0.05; *n* = 3). Similar results were observed in transwell migration assays (Fig. 4C; *P*<0.05; *n* = 3). We examined the actin stress fibers and focal adhesion complex formation by staining with FITC-phalloidin (actin filaments) and anti-vinculin (focal adhesions). Fig. 4D shows similar expression and localization between TSP1+/+ and TSP1−/− ChEC. This was further confirmed by measuring fluorescence intensities using Image J (not shown).

### TSP1−/− ChEC Were Less Adherent

**Figure 5:**
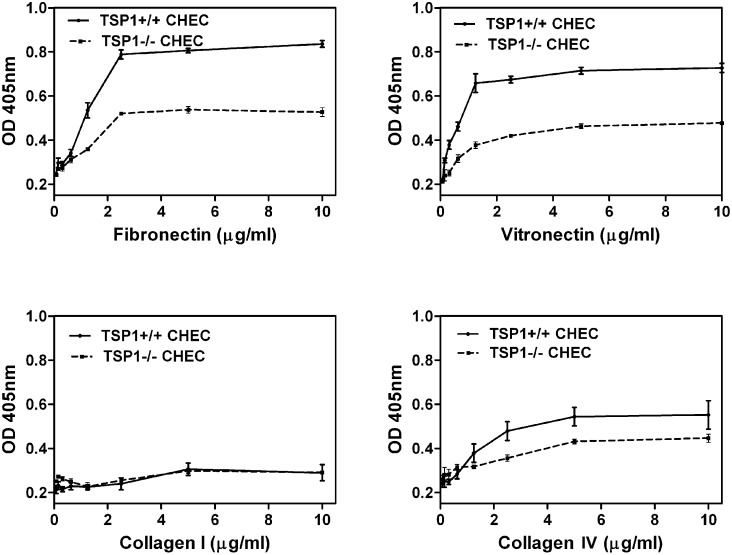
Decreased adhesion of TSP1−/− ChEC to various extracellular matrix proteins. Adhesion of ChEC to fibronectin, vitronectin, collagen I, and collagen IV was determined as described in Methods. Please note the reduced adhesion of TSP1−/− ChEC to fibronectin, vitronectin and collagen IV. None of the cells adhered to collagen I. These experiments were performed at least twice with two different isolations of choroidal EC. OD: optical density.

The defect in migration of TSP1−/− ChEC suggested alteration in their adhesion properties. We next examined the adhesion of TSP1+/+ and TSP1−/− ChEC to various extracellular matrix (ECM) proteins. Fig. 5 shows that TSP1−/− ChEC adhered less to fibronectin, vitronectin, and collagen IV compared with TSP1+/+ ChEC. Neither TSP1+/+ nor TSP1−/− cells adhered well to collagen I. Thus, TSP1 deficiency had a significant impact on adhesion of ChEC to various ECM proteins, and it is consistent with their reduced migration and increased rate of apoptosis.

**Figure 6:**
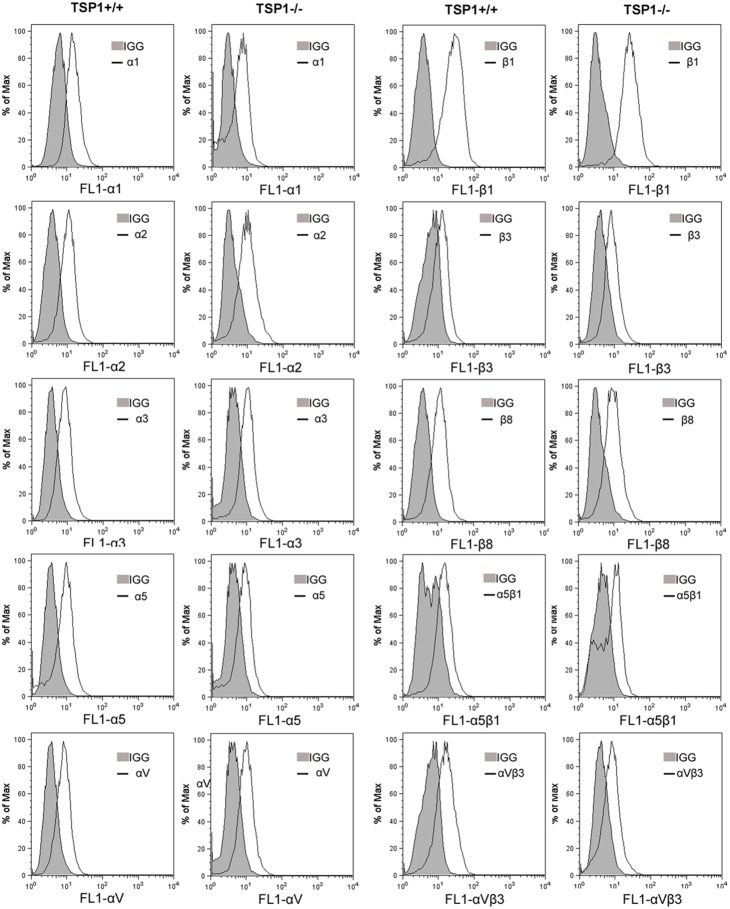
Expression of integrins in ChEC. α_1_-, α_2_-, α_3_-, α_5_-, α_v_-, β_1_-, β_3_-, and β_8_-integrin expression of ChEC was determined by FACS analysis as described in Methods. The shaded graphs show staining in the presence of control IgG. Please note the similar expression for α_1_-, α_2_-, α_3_-, α_5_-, α_v_-, β_1_-, β_3_-, and β_8_-integrin in TSP1+/+ and TSP1−/− ChEC. The levels of α_5_β_1_- and α_v_β_3_-integrin were decreased in TSP1−/− ChEC. These experiments were repeated with two different isolations of cells with similar results.

In an attempt to determine whether the altered adhesive properties are due to changes in expression and/or activity of integrins on ChEC, we examined the expression of various integrins by FACS analysis. The expression levels of α_1_-, α_2_-, α_3_-, α_5_-, α_v_-, β_1_-, β_3_-, and β_8_-integrins showed no significant differences between TSP1+/+ and TSP1−/− ChEC (Fig. 6). However, TSP1−/− ChEC showed an approximately 50% decrease in the level of α_5_β_1_- and α_v_β_3_-integrins, consistent with their decreased adhesion to fibronectin, vitronectin, and collagen IV.

### Expression of ECM Proteins by ChEC

**Figure 7:**
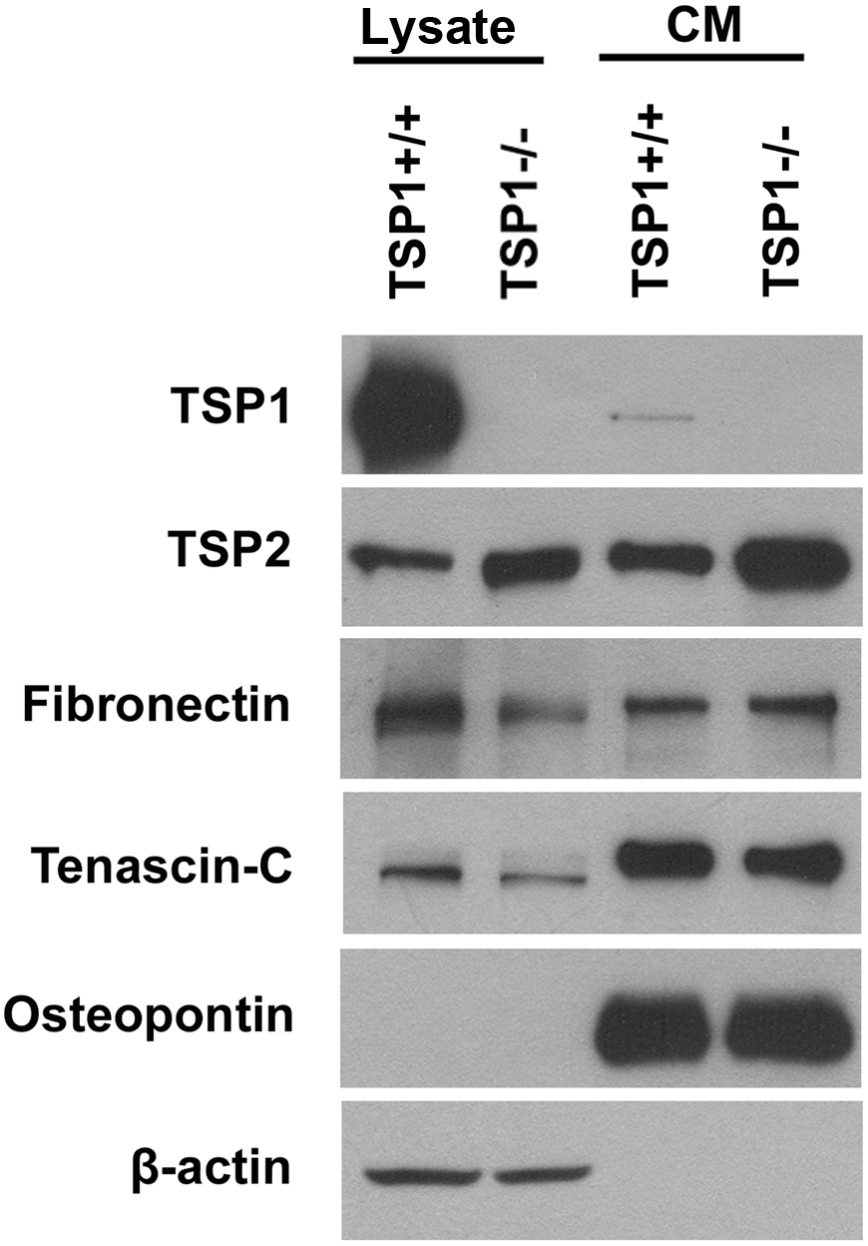
Altered expression of ECM proteins in TSP1−/− ChEC. TSP1+/+ and TSP1−/− ChEC were incubated for 2 days in serum-free medium. The collected conditioned medium (CM) and cell lysates were analyzed by Western blotting for TSP1, TSP2, fibronectin, tenascin-C, and osteopontin using specific antibodies. These experiments were repeated with two different isolations with similar results. Please note the lack of TSP1 expression but increased TSP2 expression in TSP1−/− ChEC. Similar expression of fibronectin, tenascin-C and osteopontin was observed in TSP1+/+ and TSP1−/− ChEC.

TSP1 is a matricellular protein and a potent endogenous inhibitor of angiogenesis with a significant impact on EC proangiogenic properties [44]. We next examined the TSP1 expression in TSP1+/+ and TSP1−/− ChEC by Western blot analysis of the conditioned medium and cell lysates. Fig. 7 shows that TSP1+/+ ChEC produce a significant amount of cell associated TSP1 with lower amounts in the conditioned medium. However, the TSP1−/− ChEC did not produce TSP1, as expected (Fig. 7). TSP2, a closely related family member with antiangiogenic activity, was detected in cell lysates and conditioned medium prepared from ChEC. However, the TSP2 level was increased in TSP1−/− ChEC, perhaps compensating for the absence of TSP1. Fibronectin, tenascin C, and osteopontin are major components of the ECM and play important roles in cell migration, wound repair, and inflammation [45]–[47]. TSP1−/− ChEC produced lower levels of fibronectin and tenascin-C, but similar levels of osteopontin compared to TSP1+/+ cells (Fig. 7).

### Attenuation of Capillary Morphogenesis in TSP1−/− ChEC

**Figure 8:**
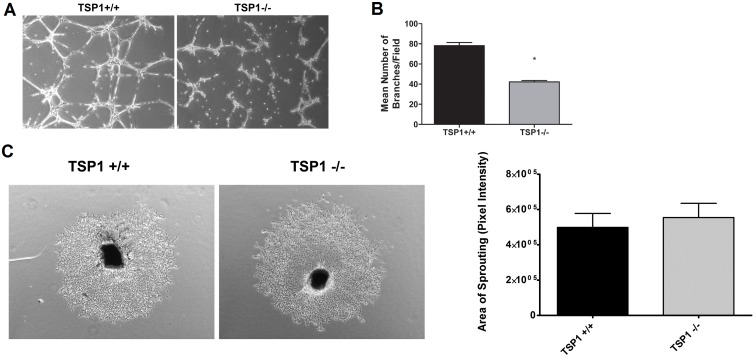
Attenuation of capillary morphogenesis of TSP1−/− ChEC. *A*: TSP1+/+ and TSP1−/− ChEC were plated on Matrigel, and capillary morphogenesis was monitored for 3 days. The photographs were taken in digital format after 18 h when optimal capillary morphogenesis was observed. *B*: Quantification of the mean number of branch points from 5 high-power fields (×100). Please note a significant decrease in capillary morphogenesis of TSP1−/− ChEC compared with TSP1+/+ cells (*P<0.05; *n* = 5). These experiments were repeated with two different isolations of choroidal EC with similar results. *C*: Choroidal ex-vivo sprouting of P21 TSP1+/+ and TSP1−/− mice. Choroidal-RPE explants were prepared and cultured as described in Methods. Images shown here represent results obtained from three animals per genotype (x40). *D*: The quantitative assessment of sprouting data showed an increase in sprouting of TSP1−/− samples but it did not reach significant levels.

Angiogenesis is led by migration and capillary morphogenesis of EC. The ability to form capillary-like structures is an important feature of EC distinguished from other cell types. Most EC form and organize into a capillary-like network in Matrigel. We investigated whether TSP1 expression affects capillary morphogenesis of ChEC. Fig. 8A shows TSP1+/+ ChEC formed a well-organized capillary-like network in Matrigel. However, the ability of TSP1−/− ChEC to form a capillary-like network was attenuated. A quantitative assessment of the data is shown in Fig. 8B (*P<*0.05; *n* = 10).

We have previously shown that the mice deficient in TSP1 exhibit enhanced choroidal neovascularization (CNV) in the laser-induced CNV model, and this was mainly attributed to the enhanced accumulation of macrophages at the sites of lesions [48]. We next asked whether lack of TSP1 affects the ex vivo sprouting of RPE-choroid complex. We observed no significant differences in ex vivo sprouting of RPE-choroid complex prepared from TSP1−/− mice compared to TSP1+/+ mice (Fig. 8C). These results further support the important role of macrophages in choroidal vascular homeostasis and neovascularization in vivo.

**Figure 9:**
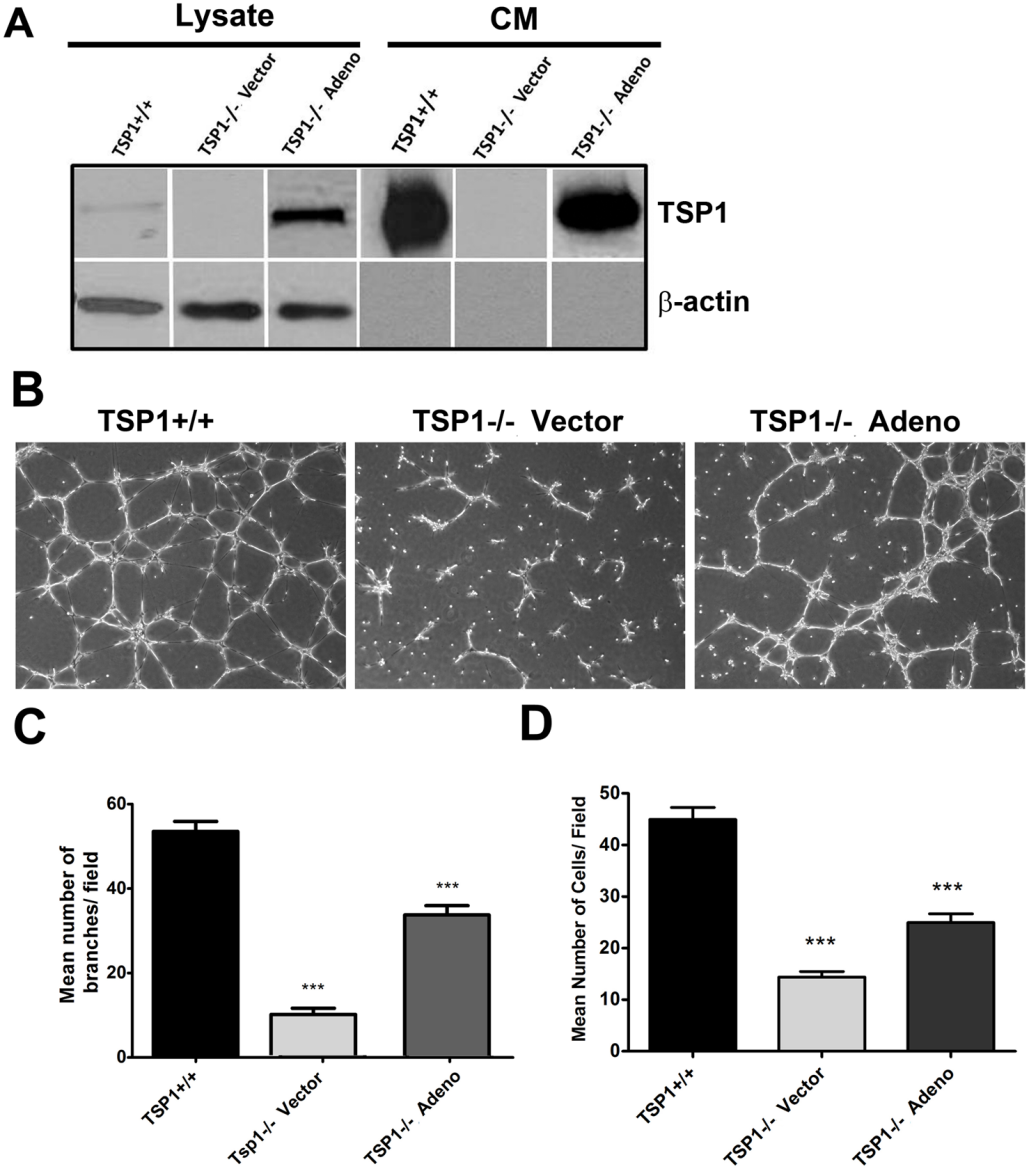
The re-expression of TSP1 in TSP1−/− ChEC. *A* : TSP1−/− cells were infected with viruses encoding TSP1 as detailed in Methods. The expression of TSP1 was confirmed by Western blot analysis. *B*: The capillary morphogenesis of TSP1−/− ChEC expressing TSP1 in Matrigel. Please note the restored capillary morphogenesis of TSP1−/− ChEC after re-expression of TSP1. *C*: The quantitative assessment of capillary morphogenesis. Please note a significant increase in the capillary morphogenesis of TSP1−/−l ChEC expressing TSP1 (***P<0.001, n = 3). *D*: Restoration of migration in TSP1−/− ChEC cells expression TSP1, determined by transwell migration assay (***P<0.001, n = 3).

We next asked whether expression of TSP1 is sufficient to restore the migratory and capillary morphogenesis of TSP1−/− ChEC. We showed re-expression of TSP1 was sufficient to restore capillary morphogenesis (Fig. 9B, C) and migration (Fig. 9D) of TSP1−/− ChEC. Thus, lack of TSP1 has a significant impact on migration and capillary morphogenesis of ChEC, which may be influenced by the presence of RPE cells and/or macrophages under ex vivo and in vivo conditions.

### Increased Phosphorylation of eNOS and NO Production in TSP1−/− ChEC

**Figure 10:**
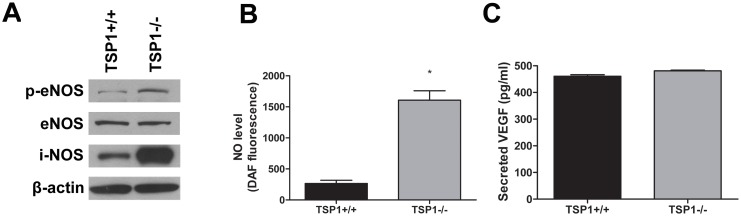
Alterations in expression and activation of NOS in ChEC. *A*: The level phosphorylated-eNOS (p-eNOS) and total eNOS, iNOS, and nNOS (not shown) in cell lysates were analyzed by Western blotting. The β-actin was used for loading control. Please note a significant increase in the level of p-eNOS and iNOS in TSP1−/− ChEC compared with TSP1+/+ cells (*P<0.05; n = 3). This was confirmed by measuring band intensities relative to β-actin, and we did not detect nNOS in both cell types (not shown). *B*: intracellular nitric oxide (NO) level in ChEC was measured using 4-amino-5- methylamino-2,7-difluorofluorescein (DAF-FM) as described in Methods. Please note a significant increase in intracellular NO level in TSP1−/− ChEC compared with TSP1+/+ cells (*P<0.05; *n* = 3). *C:* secreted level of VEGF in ChEC was determined using an ELISA immunoassay as described in Methods. Please note the similar level of VEGF secretion in ChEC (*P*>0.05; *n* = 3). These experiments were repeated with two different isolations of cells with similar results.

VEGF is a major player in retinal vascular development and angiogenesis [49], [50]. A major pathway used by VEGF to promote angiogenesis is through activation of endothelial nitric oxide synthase (eNOS) [51]–[53], and TSP1 inhibits NO mediated angiogenesis [54]. We next determined the expression and phosphorylation level of eNOS, inducible NOS (iNOS) and neuronal NOS (nNOS) (the three known NOS isoforms) in TSP1+/+ and TSP1−/− ChEC by Western blot analysis. Compared with TSP1+/+ ChEC, TSP1−/− ChEC expressed more phosphorylated eNOS, but similar levels of total eNOS. TSP1−/− ChEC also showed a significant increase in iNOS expression compared with TSP1+/+ ChEC (Fig. 10*A*). We were unable to detect nNOS expression in TSP1+/+ and TSP1−/− ChEC (not shown). We next determined NO levels in TSP1+/+ and TSP1−/− ChEC. Fig. 10B shows a significant increase (6-fold) in the level of intracellular NO produced in TSP1−/− ChEC (*P*<0.05; *n* = 3). However, TSP1+/+ and TSP1−/− ChEC produced similar levels of VEGF (Fig. 10*C*; *P*>0.05; *n* = 3).

### The Status of Src/PI3K/Akt and MAP Kinase Signaling Pathways in ChEC

**Figure 11:**
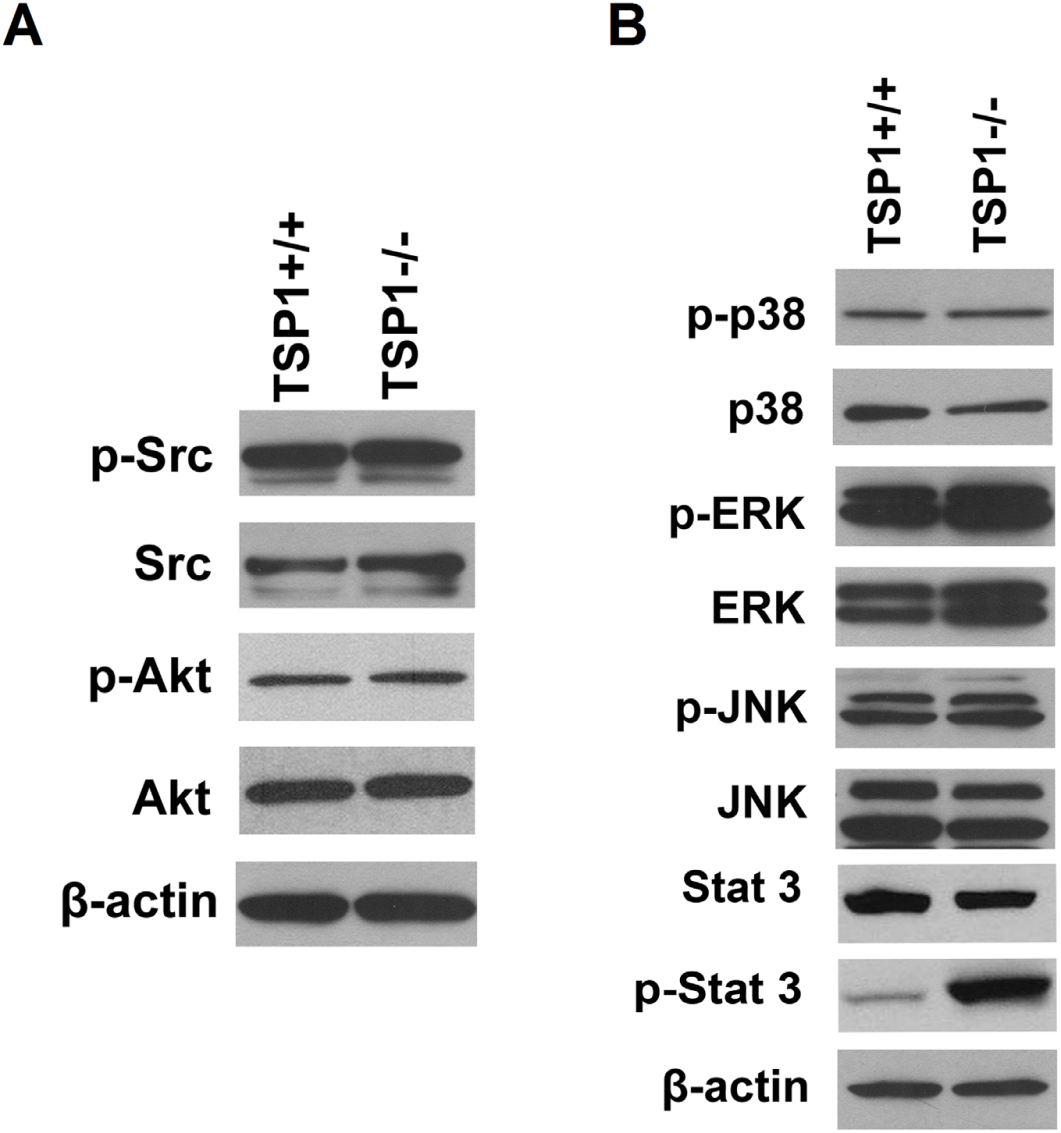
Expression and phosphorylation of Src, Akt, and MAPKs signaling pathways in ChEC. Expression and phosphorylation of Src and Akt were analyzed by Western blotting (*A*). A similar level of phosphorylated and total Src and Akt was observed in TSP1+/+ and TSP1−/− choroidal EC. Expression and phosphorylation of ERKs, JNK and p38 MAP kinases were analyzed by Western blotting (*B*). Please note minimal impact of TSP1-deficincy on phosphorylation and expression of ERKs in ChEC. A significant increase in phosphorylation of STAT3 was observed in TSP1−/− ChEC, while total level of STAT3 was not affected. These experiments were repeated with two different isolations of cells with similar results.

The Src/PI3K/Akt and MAPK signaling pathways play pivotal roles in proliferation and migration of EC including ChEC [11], [55]. We determined the expression of phosphorylated and total level of Src, and Akt in TSP1+/+ and TSP1−/− ChEC by Western blot analysis. We observed minimal changes in the levels of phosphorylated and total Src and Akt in TSP1+/+ and TSP1−/− ChEC (Fig. 11A). The activation status of MAP kinases including ERKs, JNK and p38 in TSP1+/+ and TSP1−/− ChEC were assessed by Western blotting using phospho-specific and total protein antibodies. The phosphorylated and total level of ERKs, P38, and JNK MAPK were not dramatically affected in the absence of TSP1. However, we observed a significant increase in expression of pSTAT3 in TSP1−/− ChEC compared with TSP1+/+ cells (Fig. 11B). These results are consistent with the pro-inflammatory phenotype of TSP1−/− ChEC, and are concomitant with the increased oxidative sensitivity, increased VEGF-R1 and iNOS expression in these cells.

## Discussion

Here we report the successful isolation and culture of ChEC from TSP1+/+ and TSP1−/− mice. Culture of ChEC from genetically modified mice will allow us to gain a more detailed understanding of the functional consequences that specific genes have on choroidal endothelium homeostasis. Previous preparation of ChEC from mice has been difficult and tedious, and not reported. The isolation of ChEC from choroidal tissue is complicated and labor intensive because of the small size of the choroid and the difficulty of excluding contaminating cells. We report a method for routine isolation and propagation of ChEC from mice. The magnetic beads coated with antibodies against the endothelial cell specific marker PECAM-1 were used to enrich for ChEC. The immortomouse expresses a thermolabile strain (tsA58) of the simian virus (SV) 40 large T antigen (taA58 Tag) driven by an inducible major histocompatibility complex H-2K promoter, thus eliminating many intrinsic problems with immortalized lines [56]. The T antigen expression is functionally evident at the reduced temperature of 33°C and enhanced in the presence of interferon-γ. Generally, incubation at 37°C in the absence of interferon-γ results in loss of large T antigen by 48 h [56]. We showed successful isolation and culture of ChEC from TSP1+/+ and TSP1−/− mice. FACScan analysis showed nearly all of the isolated cells express PECAM-1, VE-cadherin and B4-lectin.These cells were readily passaged and propagated in culture for up to six months without significant loss in expression of EC markers. However, these cells showed undetectable levels of PV-1 and HARA (stibilin-2), markers of fenestrated EC [57]–[59]. These observations are consistent with very limited degree of fenestration detected in these cells by electron microscopy examination (not shown). To our knowledge, this is the first report of isolation and culture of ChEC from wild type and transgenic mice.

The ability to culture ChEC from TSP1−/− mice allowed us to delineate the cell autonomous effects of TSP1 deficiency on angioinflammatory phenotype of these cells. Our laboratory was also first to report the successful culture of retinal EC from wild type and transgenic mice using a similar strategy [34]. Our previous results showed that the wild type and TSP1−/− retinal EC also exhibit similar morphology [34] as we demonstrated here for ChEC. However, the impact of TSP1-deficiency on retinal EC phenotype was significantly different from those reported here for ChEC. Retinal EC prepared from TSP1−/− mice were more migratory [34], [60], while TSP1−/− ChEC were less migratory. In addition, lack of TSP1 minimally affected retinal neovascularization during oxygen-induced ischemic retinopathy, while significant enhancement of neovascularization was observed during laser-induced choroidal neovascularization [25], [48]. These results are consistent with different proteomic profiles of human retinal and choroidal EC, especially in regards to proteins involved in regulation of angiogenesis [13].

We also observed a decreased rate of proliferation in TSP1−/− ChEC which was mainly attributed to a decreased level of DNA synthesis and increased level of apoptosis. This is in contrast to what we reported in retinal EC, where we showed TSP1−/− retinal EC grow faster compared with TSP1+/+ retina EC [60]. The TSP1−/− ChEC’s ability to form capillary-like structures in Matrigel was severely compromised, while wild type ChEC formed extensive network of capillaries on Matrigel similar to retinal EC, which are able to organize regardless of TSP1 status [34]. These differences in TSP1 function in the retina vs. choroid further demonstrate the significant differences among EC of different vascular beds and their tissue specific functions. Previous studies have also shown differences in gene expression profiles and responses to various cytokines between choroidal and retinal EC including responses to high glucose and VEGF isoforms [10], [13]. Identification of such differences will help to understand tissue specific vascular functions and their vascular bed specific therapeutic targeting.

TSP1−/− ChEC were less adherent on fibronectin, vitronectin, and collagen IV compared with TSP1−/− ChEC. The alteration in the adhesion of TSP1−/− cells was attributed, at least in part, to the changes in expression of specific integrins and ECM proteins. Although an increase in TSP2 level was observed in TSP1−/− ChEC, it was not sufficient to restore defects in the proliferation and migration of TSP1−/− ChEC. Thus, TSP1 plays an important role in ChEC proliferation and migration which cannot be compensated for by an increase in TSP2 expression.

The expression of VE-cadherin is thought to be specific to vascular EC and generally used as a marker of mesenchymal precursor cells that may develop into vascular EC and/or hematopoietic cells. FACScan analysis showed a similar expression of VE-cadherin in TSP1+/+ and TSP1−/− ChEC. However, the VE-cadherin expressed in these cells did not appear to localize to sites of cell-cell contact, as it does in retinal EC [34], despite using VE-cadherin antibodies from multiple sources. The reason for this lack of VE-cadherin junctional localization and/or detection in Western blots is not clear, and may be due to absence of adherens junctions in ChEC and/or antibody specificity. In contrast, the other major EC cadherin, namely N-cadherin, was abundantly expressed in ChEC and showed junctional localization. Thus, the formation of adherens junctions and its components in ChEC require further study.

Endoglin (CD105) is a membrane protein involved in the TGF-β receptor signaling pathway with predominant expression in proliferating endothelial cells [61]. We have observed significant up-regulation of endoglin in retinal vasculature during oxygen-induced ischemic retinopathy when retina undergoes active neovascularization, and its deficiency results in attenuation of retinal neovascularization and proangiogenic activity of retinal EC [39]. We observed very low expression of endoglin in TSP1+/+ ChEC, and was undetectable in TSP1−/− ChEC. This is consistent with Grisanti et al who found that not all vascular EC in choroidal neovascular membranes express endoglin, and endoglin expression was rarely associated with proliferating Ki-67 positive EC [62]. These observations are also consistent with similar degree of choroidal neovascularization in endoglin-deficient mice in a mouse model of laser-induced choroidal neovascularization (our unpublished results). Thus, endoglin expression and/or function in choroidal angiogenesis may be minimal.

VEGF signaling through its receptor (VEGFR-2) results in activation of Akt1 and its downstream cell protective events, which may be influenced by the levels of VEGF-R1. The endothelial NOS is a downstream target of Akt1 and its phosphorylation by Akt1 results in its activation and production of NO and VEGF-mediated angiogenesis. TSP1 inhibits NO mediated angiogenesis in a cGMP dependent and independent manner [54]. In addition, decreased levels of VEGF-R1 is associated with decreased Akt and eNOS phosphorylation and iNOS activity perhaps through modulation of STAT3 activity [63]. Choroidal EC fromTSP1−/− mice expressed increased level of phosphorylated (active) eNOS and a significant increase in intracellular NO level compared with TSP1+/+ ChEC. In addition, TSP1−/− ChEC expressed significantly higher levels of iNOS, a marker of inflammation, which can produce significant amounts of NO and oxidative stress [64]–[66]. This is consistent with the proinflammatory phenotype of TSP1−/− mice when exposed to laser-induced choroidal neovascularization and enhanced neovascularization [48]. Although the changes in phosphorylated eNOS and increased iNOS expression/activity and NO level were independent of changes in Akt1 expression and/or activation, we observed increased levels of VEGF-R1 in TSP1−/− ChEC. Thus, in the absence of TSP1 the expression and/or activity of phosphorylated eNOS and increased NO level may be uncoupled from Akt1 activation and mainly attributed to increased STAT3 activity and expression of iNOS, since iNOS is most efficient NOS for production of NO and vascular dysfunction [64]–[66]. The details of these possibilities are currently under investigation in our laboratory.

In summary, we described a simple method for the isolation and culture of ChEC from TSP1+/+ and TSP1−/− mice. These cells readily propagated at permissive temperature and retained their EC characteristics in long-term cultures. We showed a significant impact for lack of TSP1 on ChEC cell-cell and cell-matrix interactions, proliferation, migration, capillary morphogenesis, and phosphorylated eNOS, iNOS expression/activity and NO production. The potential contribution of increased VEGF-R1 expression and STAT3 activity to these events, in the absence of TSP1, needs further investigation. These cells will help to advance our understanding of the regulatory mechanisms which keep ChEC in check and how their alterations, such as changes in TSP1 level, may contribute to the pathogenesis of many diseases including exudative AMD.
